# Biofilms and antibiotic resistance profile of *Enterococcus faecalis* in selected dairy cattle farm environments in Bangladesh

**DOI:** 10.1371/journal.pone.0323667

**Published:** 2025-05-19

**Authors:** Naeem Ahammed Ibrahim Fahim, Md. Liton Rana, Md Abdullah Evna Hasan, Samia Salam, Rony Ibne Masud, Nazmul Huda, Sukumar Saha, Md. Tanvir Rahman

**Affiliations:** 1 Department of Microbiology and Hygiene, Faculty of Veterinary Science, Bangladesh Agricultural University, Mymensingh, Bangladesh; 2 National Engineering Research Center of Industrial Wastewater Detoxication and Resource Recovery, Chinese Academy of Sciences, Beijing, China; 3 University of Chinese Academy of Sciences, Beijing, China; 4 Bangladesh Livestock Research Institute, Savar, Dhaka, Bangladesh; Cornell University, UNITED STATES OF AMERICA

## Abstract

Enterococci are opportunistic zoonotic pathogens. Dairy cattle and farm environments are considered important sources of *Enterococcus* spp. Here, we detected biofilm-forming *Enterococcus faecalis* circulating in dairy cattle and farm environments, followed by the detection of their virulence genes, antibiogram phenotype analysis, and genotype characterization. Isolates were cultured and identified by PCR. Ability to biofilm formation was assessed using the Congo red agar test., followed by a disk diffusion test for antibiogram and PCR for virulence and resistance genes detection. Among 150 samples collected from 12 farms, 145 were culture-positive for Enterococci. Among these, 74 were PCR screened, of which 54.05% (40/74, CI 95%: 42.78–64.93) were *E. faecalis*. About 50% of *E. faecalis* isolates were strong biofilm formers, 37.5% were intermediate, and 12.5% were weak biofilm formers. In the antibiogram study, 87.5% of isolates were resistant to rifampicin, 75% to erythromycin, 67.5% to vancomycin, and 62.5% to ampicillin. Of the positive isolations of *E. faecalis*, 80% were positive for the *vanA* gene, and 50% were positive for the *blaTEM* resistance gene. Surprisingly, about 70% (28/40) of isolates showed a multidrug resistance phenotype. The Highest levels of multidrug-resistant *E. faecalis* were present in manure (87.5%) and isolates from Ullapara, Sirajganj. In PCR, 83.33%, 87.50%, 92.67%, 75%, 87.50%, and 58.33% isolates were positive for virulence genes *agg, ace, pil, fsrA, fsrB*, and *gelE*. This study marks the first investigation in Bangladesh focused on the molecular identification of biofilm-forming, multidrug-resistant strains of *E. faecalis* from dairy cattle and farm environments. We recommend implementing a One Health approach with the adoption of effective biosecurity and good farm management to monitor this multi-drug-resistant (MDR) *E. faecalis* in dairy cattle and farm environments, aiming to effectively tackle the critical challenge of antimicrobial resistance.

## Introduction

*Enterococcus* is a zoonotic opportunistic pathogen and *Enterococcus faecalis* and *Enterococcus faecium* are the two main species considered emerging pathogens [1]. *E. faecalis*, a Gram-positive, opportunistic, disease-causing organism in humans, is typically present in human and animal digestive tracts and is also regarded as a fecal indicator in environmental samples such as soil or water. Due to its strong survival ability, it can adapt to a diverse range of environments [2]. It is prevalent mainly in animal guts and can spread from dairy cattle to farm environments [3]. In humans, endocarditis, bacteremia, UTI, intra-abdominal, pelvic, and soft tissue infections can be caused by *Enterococcus* [4]. A National Healthcare Safety Network report demonstrated that around 40% of vancomycin and ampicillin-resistant *Enterococcus* infections are caused by medical-associated instruments such as urinary catheters and ventilators [5]. In addition, birds, fish, seafood, wild animals, and other mammals are also recognized as the sources of these organisms, which can cause human disease by eating contaminated food or by direct contact. *E. faecalis* can also be found in mastitis-affected cattle, posing a significant public health concern [6].

Organisms with biofilm formation abilities usually use this capability to escape the effects of antibiotics. These biofilms are created when microbial cells cluster together and are encircled by extracellular polymeric materials [7]. 80% of bacterial infections are associated with biofilm formation ability [8]. Enterococci can form biofilms, which contribute to antimicrobial resistance, pathogenicity, and resistance to environmental stressors. Several genes are important for biofilm development, aiding their pathogenicity and controlling harsh stressors, including *agg* (aggregation substances), *ace* (adhesion of collagen), and *pil* (adhesion on the cell surface) together with *gelE* (gelatinase), *fsrA, fsrB, fsrC, sprE,* and *cyl* [9–12]. Under control of the fsrABC two-component regulatory system, *gelE* controls cellular chain length by removing misfolded proteins from the bacterial cell surface and hydrolyzes collagen, gelatin, and small peptides, while SprE stabilizes a single active AtlA- An endogenous lytic enzyme is essential for the division of cells into daughter cells to form that is resistant to *gelE* [13]. A member of the bacteriocin family (*cyl*), cytolysin lyses eukaryotic and bacterial cells in response to quorum sensing signals [14].

Antimicrobial resistance (AMR) is a global problem that must be addressed since treatments for many diseases will become more challenging. According to WHO, every year, 4.95 million fatalities worldwide are attributed to antibiotic resistance, predicting to double to around 10 million deaths by 2050. AMR also has a negative impact on the lives of people by affecting the economy, livestock production, and the ecosystem [15]. *Enterococcus* is a pathogen of concern attributable to a high frequency of transfer of resistance genes to other organisms and dissemination in the environment [16]. Using pheromone signaling based on the conjugation method, they transmit antibiotic-resistance genes among other species [17]. Thus, resistant enterococci in animals raised for food production—particularly vancomycin-resistant enterococci—have become a serious problem [18]. Notably, from 2000 to 2022, the trend of drug resistance among *E. faecalis* strains rate shifted over time in relation to specific drugs [19]. In Bangladesh, the prescription of antibiotics without the permission of a recognized authority is widespread. As enterococcal infections are becoming challenging to treat, a “One-health” approach is needed to combat AMR and identify the various factors that can be used to reduce it [20].

The dairy cow industry in Bangladesh is essential since it boosts employment and income levels for the people and contributes to the economy. According to the Department of Livestock Service, Bangladesh, roughly 15,00,000 cow farms are estimated to exist in Bangladesh. Dairy cattle and farm environments are significant sources of infectious agents, including pathogenic and AMR-transmitting *E. faecalis*. Previously, several studies have focused on the occurrence *E. faecalis* antibiotic resistance patterns associated with migratory birds, fish, seafood, wild animals, and mastitis-affected cattle in Bangladesh [6,20–22]. To the best of our knowledge, there is no report on the detection and characterization of biofilm-forming MDR *E. faecalis* from dairy cattle and farm environments in Bangladesh. This study aimed to fill this gap by determining the prevalence and distribution of *E. faecalis* in dairy cattle and farm environments, assessing its biofilm formation ability, detecting virulence genes, and evaluating antibiotic resistance and related genotypic traits.

## Materials and methods

### Ethical approval

The Animal Welfare and Experimentation Ethics Committee at Bangladesh Agricultural University, Mymensingh, approved the methods described in this work. [approval number AWEEC/BAU/2024(2)/20(a)].

### Collection and preparation of samples

A total of 150 samples from three distinct locations were collected, including Boyra (24.7387° N, 90.3931° E), Digarkanda (24.7589° N, 90.4077° E) (Mymensingh Sadar), and Ullapara (23.9745636° N, 89.1063788° E), (Sirajganj) between October 2023 and April 2024. Six types of samples were collected, namely feces (n = 30), floor surface (n = 30), feed (n = 24), manure (n = 30), drinking water (n = 24), and drainage water (n = 12). Samples were collected in sterile test tubes containing nutrient broth (HiMedia, Mumbai, Maharashtra, India) plugged using autoclaved cotton and transported maintaining a cool chain to the microbiology laboratory (24.7245° N, 90.4372° E), Department of Microbiology and Hygiene Department, Bangladesh Agricultural University, Mymensingh. These samples were incubated for 18–24 hours at 37°C to enhance bacterial colony formation.

### Isolation and molecular identification of *Enterococcus faecalis*

Isolation of *E. faecalis* was based on culture and Gram staining as per Rana et al., 2023 [23].

For molecular identification, DNA was extracted using a boiling method described previously [24,25]. After inoculating 50 μL of stock culture in 1 ml of nutritional broth, the mixture was incubated at 37°C overnight. After five minutes of centrifugation at 5000 rpm, the supernatant was discarded. The pellet was washed by vortexing with 500 μL of phosphate buffer solution (PBS) before centrifugation. The cell pellet was resuspended in 200 μL PBS, heated in a 100°C boiling water bath for 10 minutes, and cooled in ice for 10 minutes. Following 10-minute centrifugation at 10000 rpm, the supernatant was collected, its concentration and purity evaluated and stored for later use at -20°C.

PCR tests were conducted in a 10 µL PCR mixture containing 1 µL of forward and reverse primers presented in S1 Table, 5 µL of master mix (Promega, Madison, WI, USA), 1 µL of nuclease-free water, and 2 µL of DNA template. Following resolution on a 1.5% agarose gel and ethidium bromide staining, the PCR products were visualized using a UV transilluminator (Biometra, Göttingen, Germany). Size markers in the form of a 100 bp DNA ladder (Promega, Madison, WI, USA) were employed to verify the expected amplicon sizes.

### Determination of the capability of biofilm development in *E. faecalis*

Following established techniques, the Congo red (CR) test was used to phenotypically evaluate the enterococci’s capacity to produce biofilms [26]. Isolates were cultivated on Congo red agar (CRA) plates to test the capacity of the enterococci strains to produce biofilms. 1000 mL of blood agar (HiMedia, Maharashtra, India) was incorporated with 0.8 g of Congo red dye (HiMedia, Maharashtra, India) and 36g of sucrose (HiMedia, Maharashtra, India) to formulate the CRA plates. Sterility was ensured by incubating plates at 37°C overnight. The CRA plates were then streaked with overnight-grown enterococci cultures, which were cultured for 24–48 hours at 37°C. The features of the isolates were analyzed to ascertain their ability to form biofilms. Colonies of dusty filamentous black strains were categorized. as strong biofilm formers, while those with darkening but no dry crystalline structure w classified as intermediate/moderate. Weak biofilm formers displayed almost black colonies, and non-biofilm formers appeared as red colonies [27].

### Detection of virulence genes in *E. faecalis*

A simplex PCR method described above was used to determine the presence of virulence-related genes such as *agg, ace, fsrA, fsrB, gelE*, and *pil* in isolated *E. faecalis,* as stated in the S1 Table. Genomic DNA of *E. faecalis*, which had previously demonstrated positive results for each virulence gene, was used as PCR-positive control. For negative controls, PBS was utilized rather than genomic DNA as a template.

### Phenotypic antibiotic susceptibility test (Antibiogram testing)

Following the methods of a previous study [28], the antibiotic sensitivity pattern was determined using the disc diffusion approach according to the instructions provided by the Clinical and Laboratory Standards Institute (CLSI) 2024 (Wayne, PA, USA). To perform the antibiotic susceptibility test (AST), nine antibiotics were selected and classified according to the World Health Organization (WHO) antibiotic groups—Access, Watch, and Reserve. We focused on commercially available antibiotics (HiMedia, Mumbai, Maharashtra, India) from eight different classes: penicillins (ampicillin −10 µg/disc), amphenicols (chloramphenicol −30 µg/disc), and tetracyclines (tetracycline −30 µg/disc) were from the Access groups, glycopeptides (vancomycin −30 µg/disc), macrolides (erythromycin −15 µg/disc), fluoroquinolones (ciprofloxacin −5 µg/disc, and levofloxacin −5 µg/disc), ansamycins (rifampin -5 µg/disc) from the Watch group, and oxazolidinones (linezolid −30 µg/disc) only from the Reserve group. The bacterial colonies were cultured on EAB agar plates for 18–24 hours at 37°C in order to perform the antibiotic susceptibility test (AST). A concentration of 0.5 McFarland standard units was then achieved by suspending two to three bacterial colonies in sterile 0.85% normal saline solution. AST were performed on Mueller–Hinton agar plates.. To be classified as multi-drug-resistant (MDR), a sample had to exhibit resistance to three or more different types of antibiotics [29]. To calculate the multiple antibiotic resistance (MAR) [30] indices, the following formula was used.

$MAR\;index=\frac{The\;number\;of\;antibiotics\;to\;which\;a\;specific\;isolate\;shows\;resistance}{The\;total\;number\;of\;antibiotics}\;$

In addition, *bla*_*TEM*_ and *vanA* resistance genes were also detected using a simplex PCR method as described above with their selective primers listed in S1 Table.

### Statistical analysis

Statistical tests were conducted using GraphPad Prism version 8.4.2 (San Diego, CA, USA) and SPSS version 25 (IBM, Chicago, IL, USA). A previously described method was used to compute a binomial 95% interval (CI) using GraphPad Prism [31]. Using chi-square, differences in isolate frequencies and correlations between antibiotic resistance, virulence genes, and biofilm formation were tested. The analysis’s findings were seen as statistically significant when p < 0.05. Finally, with significance set at p < 0.05, a bivariate analysis (Z test) in SPSS was executed to investigate possible relationships between virulence genes with their antibiotic resistance pattern and biofilm formation of *E. faecalis* isolates.

## Results

### Rate of *E. faecalis* prevalence

From 150 samples, based on culture-positive traits, Gram-staining, and biochemical tests, 74 isolates were chosen and examined by PCR. 40 isolates (54.05%, 95% CI: 42.78–64.93), tested positive for *E. faecalis* by PCR (S1 Fig). The Digarkanda and Ullapara sites had the highest percentage of PCR-positive isolates, with 56.53% (13/23, 95% CI: 36.81–76.78) and 56.25% (18/32, 95% CI: 39.33–71.83), respectively, compared to Boyra, which had 47.37% (9/19, 95% CI: 27.33–68.29). In addition, among the six types, manure (72.73%, 95% CI: 43.44–90.25) and floor surface (71.43%, 95% CI: 45.35–88.28) showed higher PCR positive results than the other four samples types, drainage water (60%, 95% CI: 31.27–83.18), drinking water (46.15%, 95% CI: 23.21–70.86), feces (42.85%, 95% CI: 21.38–67.41), and feed (33.33%, 95% CI: 13.81–60.94). No statistically significant differences were found across various locations and sample types (p-value > 0.05), as presented in Table 1.

**Table 1:** Rate of detection *E. faecalis* isolates from samples of different dairy cattle and farm environments.

Categories	Locations or Samples name (N)	n (%) ^S^ [95% CI]	*p*-value
Location	Digarkanda, Mymensingh (23)	13 (56.53 ^a^) [36.81–74.37]	0.794
	Boyra, Mymensingh (19)	9 (47.37 ^a^) [27.33–68.29]	
	Ullapara, Sirajganj (32)	18 (56.25 ^a^) [39.33–71.83]	
Sample	Floor surface (14)	10 (71.43 ^a^) [45.35–88.28]	0.261
	Feces (14)	6 (42.85 ^a^) [21.38–67.41]	
	Feed (12)	4 (33.33 ^a^) [13.81–60.94]	
	Manure (11)	8 (72.73 ^a^) [43.44–90.25]	
	Drainage Water (10)	6 (60 ^a^) [31.27–83.18]	
	Drinking water (13)	6 (46.15 ^a^) [23.21–70.86]	
Total	74	40 (54.05%) [42.78 - 64.93]	

Here, within the variable being evaluated, S = values with different superscripts differ significantly (p < 0.05), N = number of isolates sampled by category, n = no of isolates for the category, CI = confidence interval

### Frequency of biofilm-formation

A large proportion of biofilm-forming isolates were determined to be strong biofilm formers in the Congo red agar test, with a 95% CI of 12.43 to 68.57 (12/24). However, 12.5% (3/24, 95% CI: 4.344–31.00) were recognized as weak biofilm formers, while 37.5% (9/24, 95% CI: 21.16–57.29) were identified as intermediate. Furthermore, the frequencies of strong, moderate, and non-biofilm-forming *E. faecalis* isolates showed a statistically significant variation, as shown in Table 2.

**Table 2:** Occurrence of biofilm-forming *E. faecalis* isolates (N = 24).

SL no	Nature of Biofilm	Occurrence of biofilm formers n (%) ^S^	95% CI (%)	*p-*value
1	Strong	12 (50 ^a^)	(31.43–68.57)	0.019
2	Intermediate	9 (37.5 ^a^)	(21.16–57.29)	
3	Weak	3 (12.5 ^b^)	(4.344–31.00)	

Here, within the variable being evaluated, S = values with different superscripts differ significantly (p < 0.05), N = number of isolates sampled by category, n = no of isolates for the category, CI = confidence interval

### Virulence genes detection

**Fig 1:**
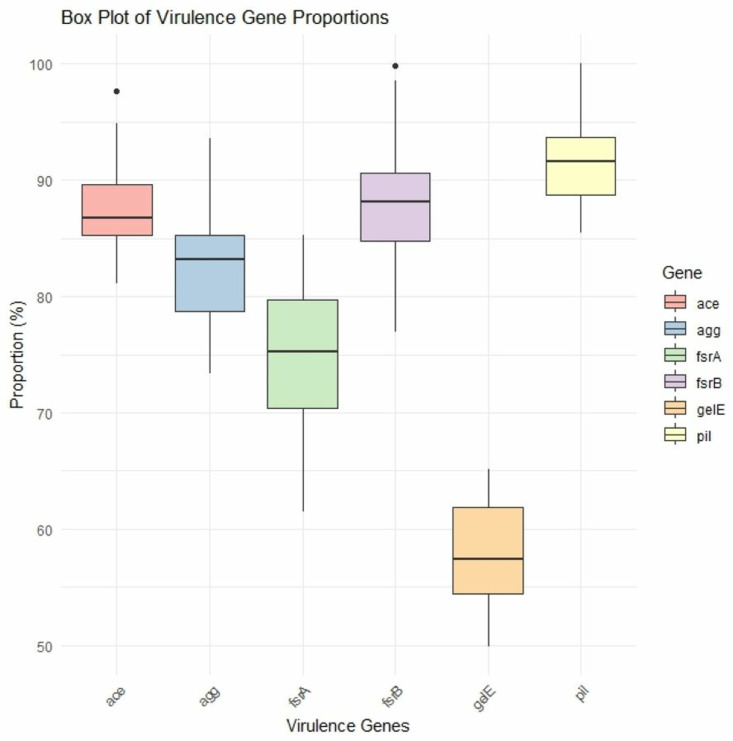
Occurrence of virulence genes in the *E. faecalis* isolate.

24 randomly selected PCR-positive isolates were subjected to molecular detection of six virulence genes. The result revealed that 20 (83.33%; 95% CI: 64.15–93.32) tested positive for the *agg* virulence gene, 21 (87.5%; 95% CI: 69.00–95.66) for *ace*, 18 (75.00%; 95% CI: 55.10–88.00) for *fsrA,* 21 (87.5%; 95% CI: 69.00–95.66) for *fsrB*, 22 (91.67%; 95% CI: 74.15–97.68) for *pil*, and 14 (58.33%; 95% CI: 38.83–75.33) for *gelE* virulence gene (Fig 1 and S1 Fig).

According to bivariate analysis, *gelE* and *fsrB* (ρ = .447) and *fsrB* and *agg* (ρ = .507) showed a strong positive and significant co-occurrence association, as shown in S2 Table and (S2 Fig).

In relation to biofilm formation, *ace* (91.7%) and *pil* (100%) genes were more common in strong biofilm formation isolates, whereas *agg* (75%), *fsrA* (75%), *fsrB* (83.3%), and *gelE* (58.3%) were more common in intermediate biofilm formation isolates, as shown in Table 3.

**Table 3:** Relationship between presence of virulence genes and biofilm formation in *E. faecalis* (N = 24) isolated from Bangladeshi dairy cattle and agricultural settings.

Virulence gene names	Biofilm formation level			Total no. of positive isolates (%) S [95% CI]	*p-value*
	Strong biofilm formers, T (%) (n = 12)	Intermediate biofilm formers, T (%) (n = 9)	Non-biofilm formers, T (%) (n = 3)		
*agg*	9 (75 ^a^)	9 (100 ^a^)	2 (66.7 ^a^)	20 (83.3 ^a, b^) [64.15–93.32]	0.223
*ace*	11 (91.7 ^a^)	7 (77.8 ^a^)	3 (100 ^a^)	21 (87.5 ^a, b^) [69.00–95.66]	0.497
*fsrA*	9 (75.0 ^a^)	7 (77.8 ^a^)	2 (66.7 ^a^)	18 (75 ^a, b^) [55.10–88.00]	0.929
*fsrB*	10 (83.3 ^a^)	9 (100 ^a^)	2 (66.7 ^a^)	21 (87.5 ^a, b^) [69.00–95.66]	0.264
*pil*	12 (100 ^a^)	8 (88.9 ^a, b^)	2 (66.7 ^b^)	22 (91.7 ^b^) [74.15–97.68]	0.162
*gelE*	7 (58.3 ^a, b^)	7 (77.8 ^b^)	0 (0 ^a^)	14 (58.3 ^a^) [38.83–75.33]	0.061

Here, Within the variable being evaluated, S = values with different superscripts differ significantly (p < 0.05), N = Total number of isolates sampled, T = number of isolates positive for each gene as biofilm formation level, n = no of isolates in each biofilm formation category, CI = confidence interval

### Antibiogram profile of *E. faecalis*

#### Overall resistance pattern of *E. faecalis.*

**Fig 2:**
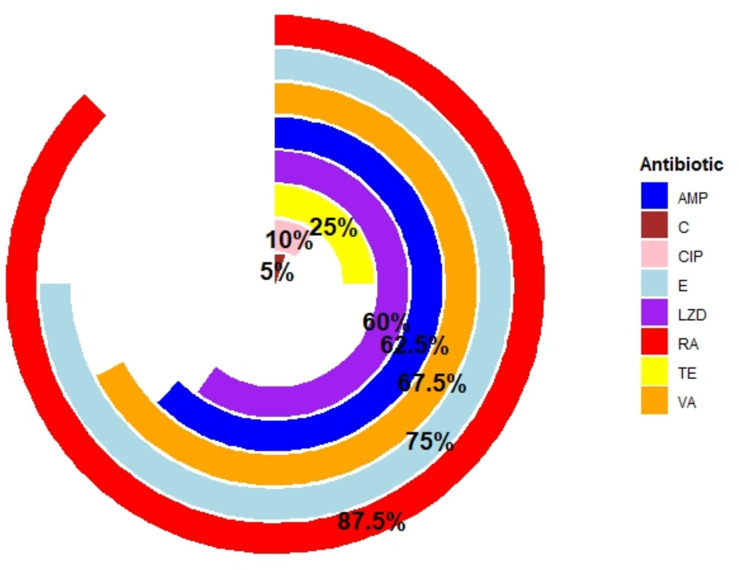
Overall resistance profile of isolated *E. faecalis.* Legends. AMP = Ampicillin; C = Chloramphenicol; LZD = Linezolid; CIP = Ciprofloxacin; E = Erythromycin; RA = Rifampicin; TE = Tetracycline; and VA = Vancomycin.

Antibiogram testing of *E. faecalis* isolates (N = 40) identified that from the WHO Watch antibiotic group, the largest number of isolates were resistant to rifampin 87.5% (35/40, 95% CI: 73.89–94.54). In addition, 75% (30/40, 95% CI: 59.81–85.81) were resistant to erythromycin, 67.5% (27/40, 95% CI: 52.02–79.92) to vancomycin, and 10% (4/40, 95% CI: 3.958–23.05) to ciprofloxacin. However, no isolates were found to be resistant to levofloxacin. For the Reserve group antibiotic, 60% of isolates (24/40, 95% CI: 44.60–73.65) showed resistance to linezolid. In the Access group, 62.5% (25/40, 95% CI: 47.03–75.78) of isolates were resistant to ampicillin, 40% (10/40, 95% CI: 14.19–40.19) to tetracycline, and 5% (2/40, 95% CI: 1.382–16.50) to chloramphenicol (Fig 2).

Moreover, 50% (5/10, 95% CI: 23.66–76.34) and 80% (8/10, 95% CI: 4902–94.33) of isolates were PCR-positive for *bla*_*TEM*_ and *vanA* (S1 Fig).

Statistical bivariate analysis revealed highly significant relationships among the antibiotic-resistant isolates analyzed in this investigation. There was a strong, positive link between the resistance patterns of vancomycin and ampicillin (ρ = 0.675; p < 0.01), tetracycline and ciprofloxacin (ρ = 0.385; p < 0.05), erythromycin and ampicillin (ρ = 0.507; p < 0.01), erythromycin and vancomycin (ρ = 0.586; p < 0.01), linezolid and ampicillin (ρ = 0.843; p < 0.01), linezolid and vancomycin (ρ = 0.850; p < 0.01), linezolid and erythromycin (ρ = 0.589; p < 0.01), rifampicin and ampicillin (ρ = 0.488; p < 0.01), rifampicin and vancomycin (ρ = 0.383; p < 0.01), rifampicin and erythromycin (ρ = 0.480; p < 0.01), and rifampicin and linezolid (ρ = 0.463; p < 0.01), as presented in S3 Table and (S3 Fig).

#### Antibiogram profiles of biofilm-forming *E. faecalis.*

When comparing the resistance profiles among the different biofilm-formation phenotypes of *E. faecalis*, the highest resistance of isolate*s* was observed in rifampin (87.5% - strong 83.3% vs intermediate 88.9% vs non-biofilm 100%) followed by erythromycin (75.00% - 75% vs 66.7% vs 100%), vancomycin (70.8% - 50% vs 88.9% vs 100%), ampicillin (62.5% - 41.7% vs 77.8% vs 100%), linezolid (62.5% - 41.7% vs 77.8% vs 100%), ciprofloxacin (25%- 25% vs 33.3% vs 0%), tetracycline (25% - 25% vs 33.3% vs 0%), and chloramphenicol (8.3% - 8.3% vs 11.1% vs 0%), as shown in Table 4.

**Table 4:** Relationship between *E. faecalis* biofilm production and antibiotic resistance pattern.

Categories	Antibiotics	Biofilm formation level			Total no. of resistant isolates (%) S [95% CI]	*p-value*
		Strong biofilm formers, T (n = 12)	Intermediate biofilm formers, T (n = 9)	Non-biofilm formers, T (n = 3)		
Phenotypic	AM	5 (41.7 ^a^)	7 (77.8 ^a^)	3 (100 ^a^)	15 (62.5) [42.71–78.84]	0.085
	VA	6 (50 ^a^)	8 (88.9 ^a^)	3 (100 ^a^)	17 (70.8) [50.83–85.09]	0.075
	CIP	3 (25 ^a^)	3 (33.3 ^a^)	0 (0 ^a^)	6 (25) [12.00–49.9]	0.513
	C	1 (8.3 ^a^)	1 (11.1 ^a^)	0 (0 ^a^)	2 (8.3) [2.31–25.85]	0.834
	TE	3 (25 ^a^)	3 (33.3 ^a^)	0 (0 ^a^)	6 (25) [12.00–49.9]	0.513
	E	9 (75 ^a^)	6 (66.7 ^a^)	3 (100 ^a^)	18 (75) [55.1 - 88.90]	0.513
	LEV	0 (0 ^a^)	0 (0 ^a^)	0 (0 ^a^)	0 (0) [0–13.8]	NA
	RA	10 (83.3 ^a^)	8 (88.9 ^a^)	3 (100 ^a^)	21 (87.5) [69.0–95.66]	0.085
	LNZ	5 (41.7 ^a^)	7 (77.8 ^a^)	3 (100 ^a^)	15 (62.5) [42.71–78.84]	0.728

Here, Within the variable being evaluated, S = values with different superscripts differ significantly (p < 0.05), N = Total number of isolates sampled, T = number of isolates for each antibiotic at each biofilm formation, n = no of isolates in each biofilm formation category, CI = confidence interval,

CIP = ciprofloxacin, TE = tetracycline, LEV = levofloxacin, RA = rifampin, P = penicillin, LZD = linezolid, AMP = ampicillin, C = chloramphenicol, VA = vancomycin, E = erythromycin, CI = confidence interval, NA = not applied

#### *E. faecalis* phenotypic MDR and MAR nature.

**The overall occurrence of multi-drug-resistant (MDR) and multiple-antibiotic resistance (MAR) *E. faecalis* isolates:** Nine resistance patterns were found in the 28 (70%, 95% CI: 54.57–81.93) isolates of *E. faecalis* that exhibited phenotypic multi-drug resistance. The most prevalent pattern, seen in 16 isolates, was AM-VA-E-LZD-RA, 57.14% (16/28, 95% CI: 39.07–73.49). Remarkably, resistance to a comparable number of antibiotics and classes was evident in all the patterns. The MAR index also fluctuated between 0.44 and 0.66, as stated in Table 5.

**Table 5:** Occurrence of *E. faecalis* isolates with multiple-drug resistance (MDR) and multiple-antibiotic resistance (MAR).

SL no	Antibiotic resistance pattern	No. of antibiotics (classes)	No. of isolates	Overall, MDR isolates %	MAR index
1	AM, VA, CIP, E, LZD, RA	6 (6)	1	28/40 (70%)	0.66
2	AM, VA, E, LZD, RA	5 (5)	16		0.55
3	AM, VA, C, TE, LZD, RA	6 (6)	1		0.66
4	AM, VA, TE, E, LZD, RA	6 (6)	5		0.66
5	VA, TE, E, RA	4 (4)	1		0.44
6	VA, CIP, TE, E, RA	5 (5)	1		0.55
7	VA, E, LZD, RA	4 (4)	1		0.44
8	CIP, C, TE, E, RA	5 (5)	1		0.55
9	AM, CIP, TE, E, RA	5 (5)	1		0.55

Here, MDR = multi-drug-resistance, MAR = multiple antibiotic resistance, AMP = Ampicillin; C = Chloramphenicol; LZD = Linezolid; CIP = Ciprofloxacin; E = Erythromycin; RA = Rifampicin; TE = Tetracycline; LEV = levofloxacin and VA = Vancomycin

#### Mapping of multi-drug-resistant (MDR) *E. faecalis* isolates.

**Fig 3:**
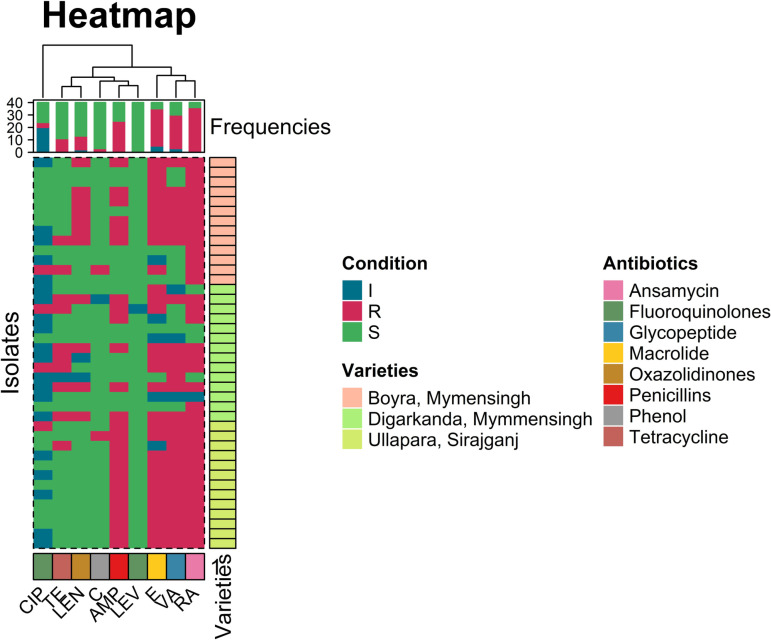
A heat map showing antibiotic resistance pattern of isolated *E. faecalis*, Legends. AMP = Ampicillin; C = Chloramphenicol; LZD = Linezolid; CIP = Ciprofloxacin; E = Erythromycin; RA = Rifampicin; TE = Tetracycline; LEV = levofloxacin and VA = Vancomycin.

Mapping of multi-drug-resistant (MDR) *E. faecalis* revealed the highest prevalence in manure (87.5%, 95% CI: 52.91–97.76) and feces (80%, 95% CI: 37.55–96.38). This was followed by floor surfaces (66.67%, 95% CI: 35.42–87.94), drinking water (66.67%, 95% CI: 30.00–90.32), drainage water (60%, 95% CI: 23.07–88.24), and feed (57.14%, 95% CI: 25.05–84.18). No statistically significant differences were observed in MDR prevalence across sample types. However, 100% of the isolates from Ullapara, Sirajganj (Fig 3), were MDR *E. faecalis*, showing a statistically significant variation in prevalence among isolates from different locations, as shown in S4 Table.

## Discussion

*Enterococcus faecalis* is a commensal organism that coexists in the gastrointestinal (GI) tract of animals, insects, birds, reptiles, and humans. Generally, it can be transmitted from animals to humans and possesses significant public health implications [32]. However, due to their ability to survive in harsh environments, biofilm formation, and transfer of resistance genes, they are a significant concern for the health sector. Moreover, dairy cattle and farm environments play a huge role in Bangladesh. As far as we know, no research has been done on the biofilm formation of *E. faecalis* isolated from dairy cattle and agricultural environments in Bangladesh or elsewhere. This work thoroughly details the virulence factors and antibiotic resistance of biofilm-forming *E. faecalis* isolated from dairy animals and agricultural settings.

### *Enterococcus faecalis* isolated from dairy cattle and farm environments

In this study, 54.05% (40/74) of isolates were positive for *E. faecalis* among the six types of samples, whereas the highest prevalence of positive isolates was from manure, 72.73%. In a similar study [33], 43.5% *E. faecalis* was found in cattle farm waste and cattle house waste in Tanzania, which was lower than in our study. However, a higher level of occurrence of *E. faecalis* in cattle was found in Portugal, about 69% [34]. Numerous factors, such as the study locations, varying environmental conditions, the variety and quantity of samples collected, and the number of bacteria present in both the samples and the regions where they were obtained, may contribute to the difference in the prevalence of *E. faecalis* isolates in dairy cattle and farm environments samples..*E. faecalis* is one of those organisms that can shed on animal feces and environmental waste and is also considered a fecal indicator [2,35]. Dairy cattle effluent can spread to human since it may have a direct connection to the nearby pond or ecosystem and has the potential to pollute human drinking water. It can also cause mastitis in dairy cows and is considered an environmental origin mastitis-causing pathogens *Enterococcus* [36]. It can be transmitted from inflamed udder to humans through raw milk. The results of this study also imply that feed and drinking water, albeit being fecal indicators, are also sources of *E. faecalis*. This suggests that humans are being contaminated when handling and feeding animals.

### Biofilm formation of *E. faecalis* isolated from dairy cattle and farm environments

Biofilm is a major issue in the medical field due to its formation on medical implants within human tissue and its involvement in numerous deadly chronic illnesses. The primary concern regarding biofilm formation is their resistance to antibiotics, which poses a challenge for therapy. A variety of physical, physiological, and gene-related factors combine to build their resistance potential [37]. These microbiological biofilm communities are created when planktonic organisms attach themselves to an abiotic surface and begin to grow. Since microorganisms are associated with ill-health, biofilm production is closely related to illnesses [38,39]. This study assessed the ability of isolated *E. faecalis* from dairy cattle and farm surroundings to produce biofilms using the CRA test. For assessing biofilm formation, the CRA test is not as sensitive as molecular and whole genome sequencing methods, but researchers nonetheless frequently utilize it due to its appropriate trade-off between sensitivity and specificity [40]. In this study, among the isolates examined, there were several biofilm producers. Of those that produced biofilms; 50% were strong producers, 37.5% were moderate, and 12.5% were weak producers. Biofilm formation during intramammary infection may aid in *E. faecalis* adhesion and colonization of the mammary gland epithelium [41]. From this, it may be concluded that biofilm formation of *E. faecalis* can move via milk from intramammary infected cows to humans, allowing the bacteria to persist in adverse environments and posing threat to human health. Little research has examined *E. faecalis*’s capacity to form biofilms in dairy cow and farm environmental samples; most have concentrated on the bacteria’s isolation and identification, virulent genes, and patterns of antibiotic resistance.

### Virulence determinants of *E. faecalis* isolated from dairy cattle and farm environments

Pathogenicity in people and animals is determined by virulent factors that are the primary cause of disease. Bacterial virulence aids colonization by enhancing bacterial adherence to host cells or adding invasive elements that promote epithelial cell invasion and weaken the immune response. In our study, about 25% of isolates harbored all virulence genes that were screened by PCR. *Pil* (91.67%), *fsrB* (87.5%), and *ace* (87.5%) were the most prevalent genes among the isolated *E. faecalis* followed by *agg* (83.33%), *fsrA* (75%), and *gelE* (58.33%). A study showed that *gelE* is a major virulence gene that helps to biofilm-formation ability in pathogenic isolates [10]. Additionally, *E. faecalis* maintained its pathogenicity by tissue adhesion and colonization by expressing the *agg* virulence gene [42]. A lot of studies examined various types of virulence genes in cattle from multiple sample types [6,41–43], but no study revealed data on virulence genes in dairy cattle and farm environments. According to the present study, there is a chance that resistant *E. faecalis* might spread from farm settings and dairy animals to humans through horizontal gene transfer, posing a risk to public health. This horizontal gene transfer has made enterococci one of the main causes of infections acquired in hospitals [44]. Additionally, enterococcal mobile genetic elements have been shown to transfer resistance determinants to more dangerous bacteria, such as *Staphylococcus aureus* [45]. In addition, Numerous virulence genes carried by *E. faecalis* that produce biofilms eventually contribute to their ability to survive in hostile environments by transferring antibiotic resistance strains through horizontal gene transfer. Our results showed that strong and intermediate biofilm-formation isolates had the highest occurrence of virulence genes compared with the weak biofilm-forming *E. faecalis* isolates. This suggests that the ability to form biofilms is related to the number of virulence genes and enhances the ability to cause disease in humans and animals. However, more thorough research is needed to identify the virulence genes and their relation to biofilm formation in dairy cattle and farm environments that may affect human health.

### Antibiogram profile of *E. faecalis* isolated from dairy cattle and farm environments

One worldwide health crisis that needs to be addressed is antimicrobial resistance. Therapy is becoming more complex every day as the misuse and mistreatment of these drugs have been linked to the issue of antibiotic resistance. Farm settings and dairy cattle are significant sources of antibiotic-resistant genes passed from animals to people. This may occur due to the management and care of animals, drainage water directly connected to adjacent water sources, and environmental discharge of livestock farm waste. Although *E. faecalis* can transmit resistance genes to humans, important in the context of little public health, little is known about the antibiotic resistance of the biofilm *E. faecalis* isolated from dairy cattle and agricultural settings. In this study, more *E. faecalis* isolates from these environments showed resistance to rifampin (87.5%) than other antibiotics. In previous research, *Enterococcus* isolates were highly resistant to rifampicin (78%) and erythromycin (48%) in animal feed [46]. However, in the present study, the most alarming finding was that 60% of our *E. faecalis* isolates were resistant to linezolid from the Reserve group. Additionally, erythromycin, vancomycin, and ampicillin resistance were found in 67.5%, 75%, and 62.5% of isolates, respectively. Since vancomycin and linezolid are last-resort medications for severe illnesses brought on by multi-drug-resistant Gram-positive bacteria, their prevalence in dairy cattle and agricultural settings poses a serious risk to public health [47,48]. The National Health Care Safety Network (NHSN) estimates that in 2006–2007, about 33 percent of all enterococci were vancomycin-resistant [5]. Additionally, vancomycin-resistant *E. faecalis* bacteremia is still linked to a higher risk of both overall hospital length of stay and in-hospital death [49]. As a result, treating the infection becomes more challenging due to this resistance. Mubita *et al* found that the greatest proportion of enterococci exhibited resistance to gentamicin, ampicillin, tetracycline, and amoxicillin [50]. Another study by A. S. Bag showed that azithromycin and tetracycline resistance were found in 40% of *E. faecalis* isolates, respectively [6]. However, Additional research employing molecular methods and MIC determination has to be carried out to obtain accurate information. In our statistical analysis, we found a significant correlation between resistance to vancomycin and ampicillin, tetracycline and ciprofloxacin, erythromycin and ampicillin, erythromycin and vancomycin, linezolid and ampicillin, linezolid and vancomycin, linezolid and erythromycin, rifampicin and ampicillin, rifampicin and vancomycin, rifampicin and erythromycin, and rifampicin and linezolid.

We also found that representatives of strong and intermediate biofilm-forming isolates were resistant to every antibiotic except levofloxacin. The highest incidence of resistance in strong biofilm formers was to rifampin, erythromycin, and vancomycin compared with ampicillin, linezolid, tetracycline, levofloxacin, ciprofloxacin, and chloramphenicol. This study suggests that biofilm forming *E. faecalis* isolates can be resistant to more antibiotics using their biofilm matrix thus providing a link between antibiotic resistance and biofilm formation. It is currently thought that biofilms are the source of more than 80% of chronic infectious disorders and that standard antibiotic treatments are unable to eradicate these biofilm-mediated infections.[51].

The use of antibiotics in agriculture has led to a significant rise in the worldwide public health problem of antimicrobial resistance in recent decades, which may have an impact on the management of human illnesses that call for antibiotic intervention. Thus, it leads to an organism being resistant to multiple antibiotics. In our study, we found 70% of isolates showed resistance to at least ≥3 antimicrobial agents and ≥3 antimicrobial classes (MDR). The common pattern of MDR is AM-VA-E-LZD-RA. Previously, 1.2% and 71% *E. faecalis* were isolated from farm animals, and their product showed resistance to at least 3 antibiotics [52,53]. Furthermore, the range of the multiple-antibiotic resistance index was 0.44 to 0.66. The MAR index can be used to infer the prudent use of antibiotics in dairy cattle farms [54]. Our study used the mapping of MDR *E. faecalis*, and the findings indicated that MDR *E. faecalis* is also present in feed and drinking water, feces, manure, drainage water, and floor surfaces. This suggests that external sources can also play a vital role as a significant reservoir and in transmitting MDR *E. faecalis* to animals on farms, which spread to humans-a serious concern for our lives.

Despite our results, there are some limitations, attributable to the sampling sites, sample size, and some financial challenges with the lack of crystal violet staining or spectrophotometric biofilm assays. However, a large sample with several locations could resolve this issue, and advanced research technology with whole genome sequencing will provide more data, although sometimes, in-depth sampling in farms is challenging due to farmers’ reluctance.

## Conclusion

Multi-drug-resistant *Enterococcu*s is considered a significant pathogen. We are aware of no prior research in Bangladesh on biofilm-forming, multi-drug-resistant *E. faecalis* isolated from dairy cattle and farm environments. Our study revealed a high prevalence of biofilm-forming, multi-drug-resistant *E. faecalis* in dairy cattle and farm environments in Bangladesh, including resistance to last-resort antibiotics such as vancomycin and linezolid, and the possibility of transfer to humans through direct or indirect contact. According to this research, strict oversight is necessary to lower the amount of antibiotics used in farms, and the government should put regulations into place to manage antibiotic resistance. The implications of our findings on the health of dairy cattle and environmental safety highlight the need for ongoing study and monitoring to protect public health and advance sustainable livestock farming techniques in Bangladesh.

## Supporting information

S1 Fig Agarose gel electrophoresis of (A) *ddlE* [941], (B) *pil* [620], (C) *fsrB* [428], (D) *agg* [413], (E) *gelE* [704], (F) *fsrA* [474], (G) *ace* [615], (H) *vanA* (713), and (I) *bla*_*TEM*_ [793]. In all cases, L: 100 bp size DNA marker; PC: positive control; NC: negative control; and the blank lane indicate the negative isolates, while the lanes with consistent bands of specific amplicon size indicate the positive isolates.
(DOCX)

S2 Fig Heatmap represents the correlation between two virulence genes of E. faecalis. (DOCX)

S3 Fig Correlations between the antibiotic-resistant isolates of E. faecalis. (DOCX)

S1 Table List of primers used to find target genes in this investigation. (DOCX)

S2 Table Pearson correlation coefficient in virulent genes of the isolated *E. faecalis.* (DOCX)

S3 Table Pearson correlation coefficient among the *E. faecalis* resistance isolates. (DOCX)

S4 Table Mapping of multi-drug-resistant *E. faecalis* isolates. (DOCX)
