# Supplementary material for: Biofilms and antibiotic resistance profile of Enterococcus faecalis in selected dairy cattle farm environments in Bangladesh
Source: PLoS One. 2025 May 19;20(5):e0323667. doi: 10.1371/journal.pone.0323667 (PMC12087997; doi:10.1371/journal.pone.0323667)
Supplement: S1 Fig — In all cases, L: 100 bp size DNA marker; PC: positive control; NC: negative control; and the blank lane indicate the negative isolates, while the lanes with consistent bands of specific amplicon size indicate the positive isolates. (DOCX) [file pone.0323667.s001.docx]

**Supporting figures**

**Supplementary Fig 1**


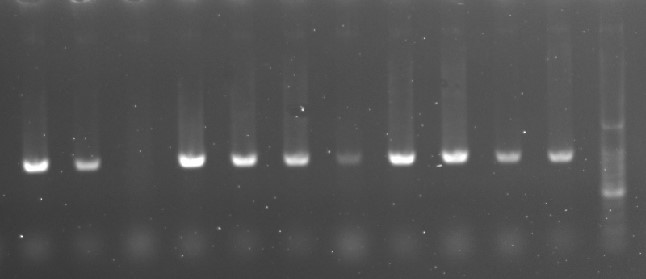


**PC 9 N 8 7 6 5 4 3 2 1 L**

**A:** Molecular detection of *ddl* gene of *Enterococcus faecalis* amplified by 941 bp. Here L represents 100 bp DNA ladder, N-negative control, PC-positive control and 1-8, and 9 represents PCR positive of isolated *Enterococcus faecalis*

941 BP


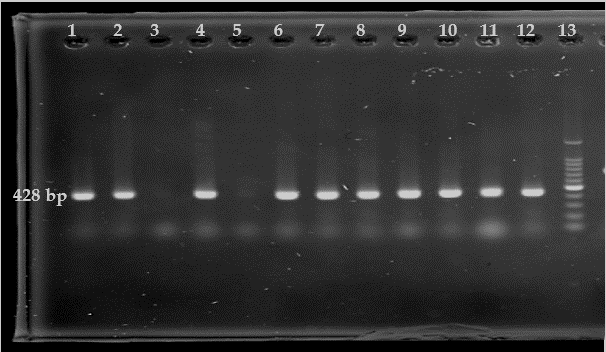

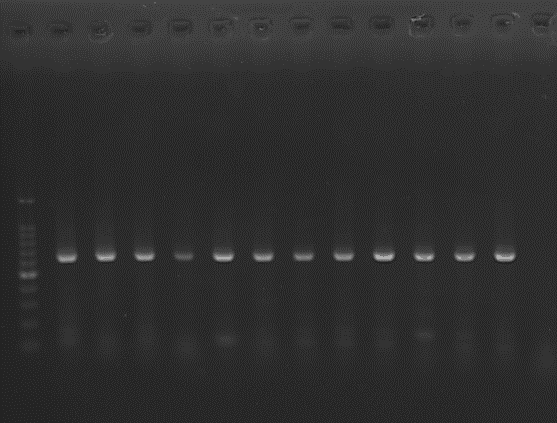


**L 1 2 3 4 5 6 7 8 9 10 11 P N**

N P 6 5 4 3 2 1 L

428 bp

620 bp

**C:** Molecular detection of *fsrB* gene that amplified by 428 bp. Here M represents 100 bp DNA ladder, N-negative control, PC- positive control, and 2- 8 represents PCR positive for *fsrB* gene

**B:** Molecular detection of *pil* gene that amplified by 620 bp. Here L represents 100 bp DNA ladder, N negative control, PC- positive control and 1-11 represented PCR positive for *pil* gene

| 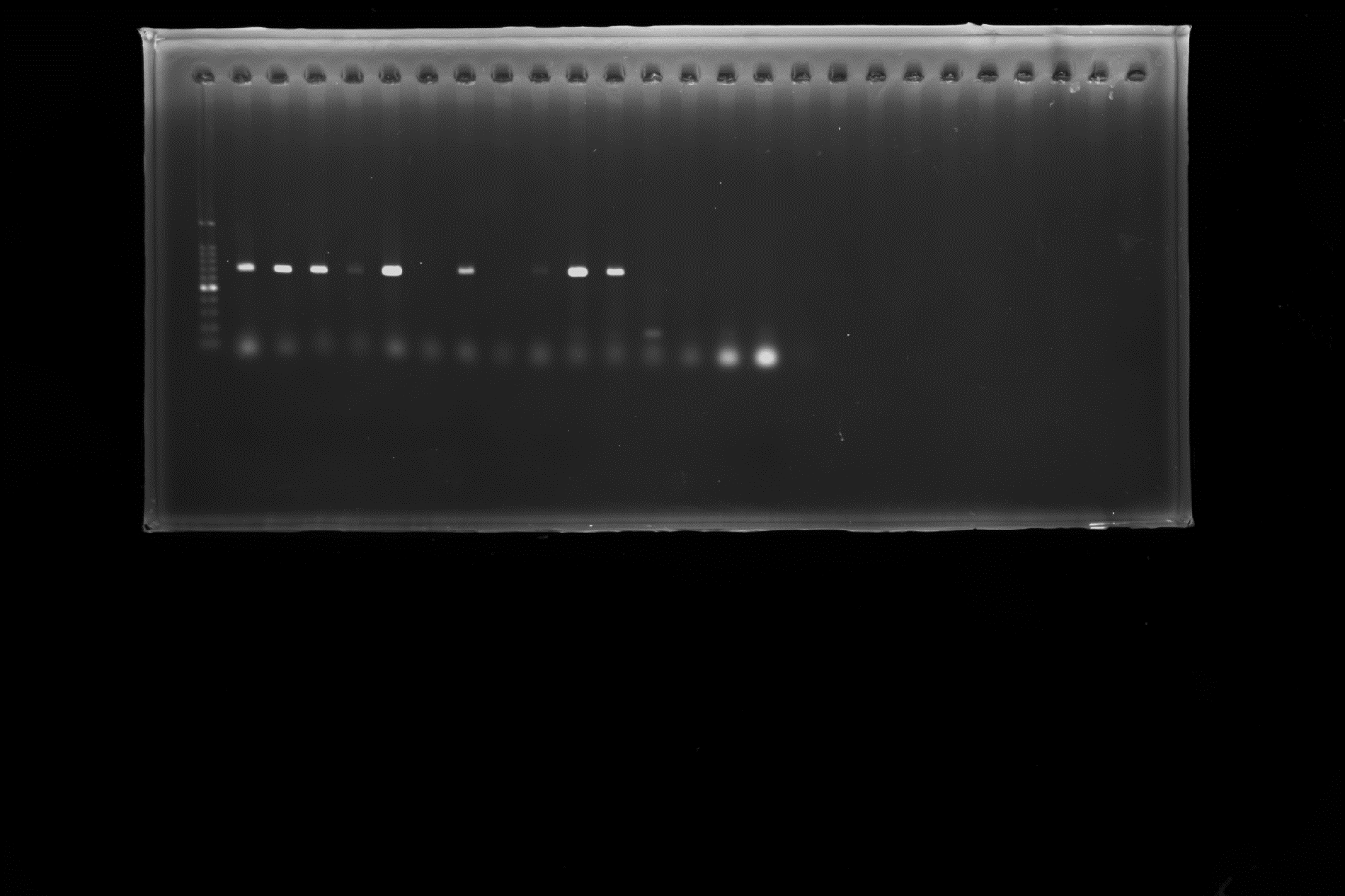 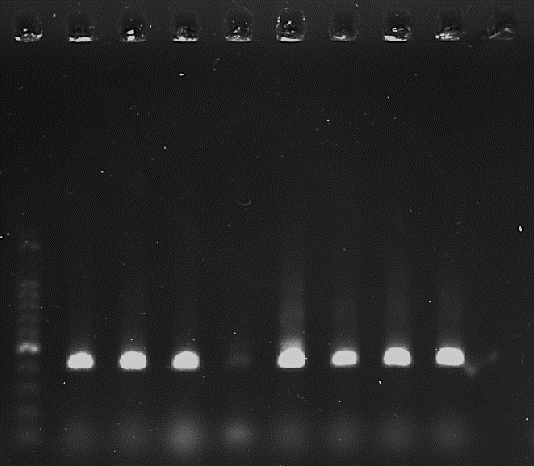 **L 1 2 3 4 5 6 7 PC N**  704 bp  **L 1 2 3 4 PC N**  413 bp    **E:** Molecular detection of *gelE* gene that amplified by 704 bp. Here L represents 100 bp DNA ladder, N negative control, P- positive control, and 1-4 represents PCR positive for *gelE* gene  **D:** Molecular detection of *agg* gene that amplified by 413 bp. Here L represents 100 bp DNA ladder, N negative control, PC- positive control, and 1-7 represents PCR positive for *agg* gene  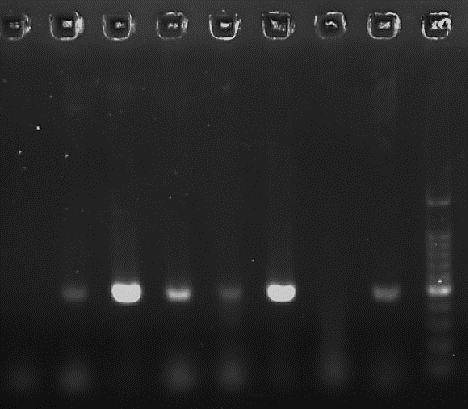  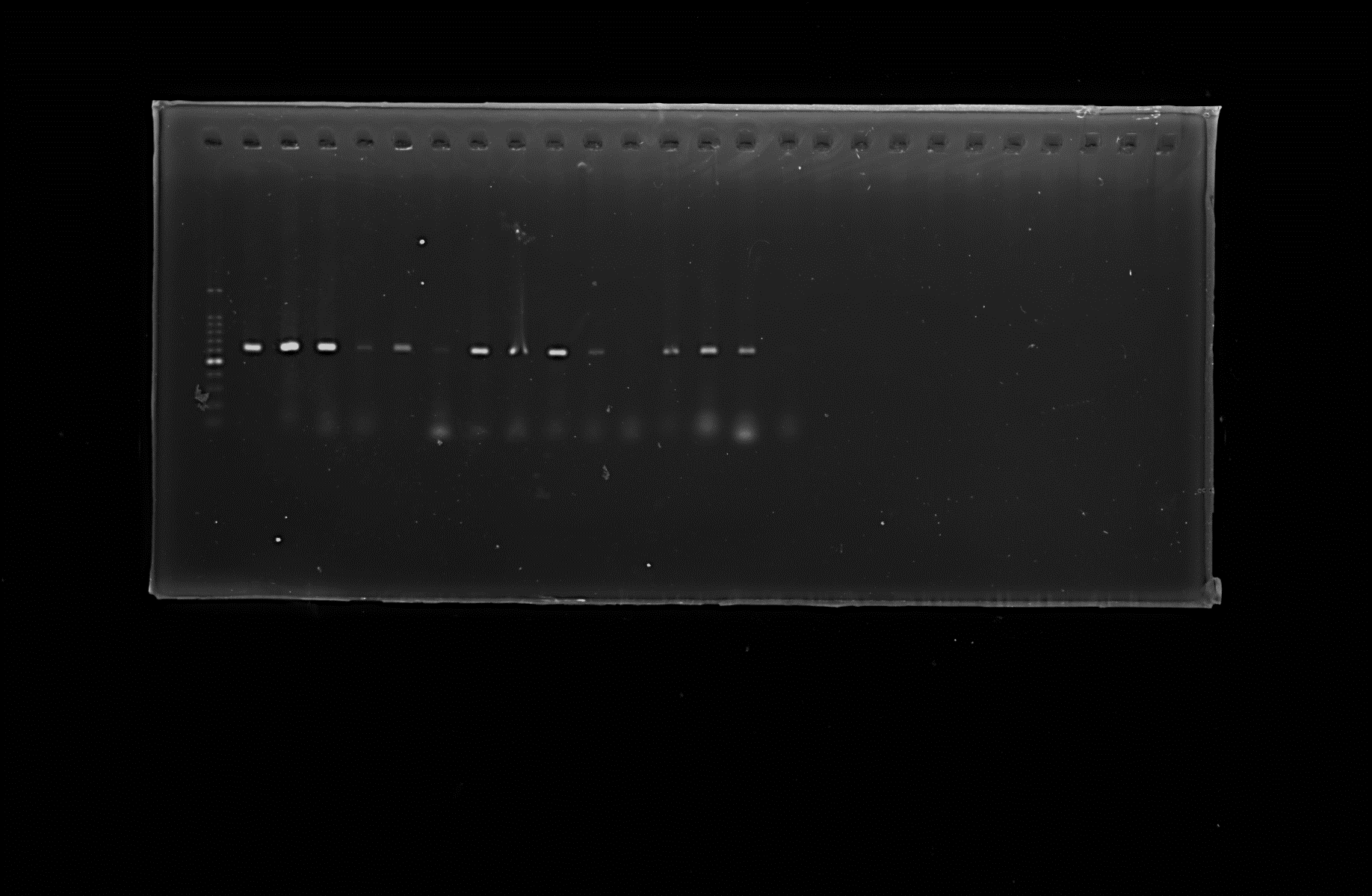  **L 1 2 3 4 5 6 7 8 9 PC N**  **PC 6 5 4 3 N 1 L**    615 bp  474 bp    **G:** Molecular detection of *ace* gene that amplified by 615 bp. Here L represents 100 bp DNA ladder, N-negative control, PC- positive control, and 1-9 represents PCR positive for *ace* gene  **F:** Molecular detection of *fsrA* gene that amplified by 474 bp. Here L represents 100 bp DNA ladder, N negative control, PC- positive control and 1, 3, 4, 5, 6 represent PCR positive for *fsrA* gene |
| --- |

| 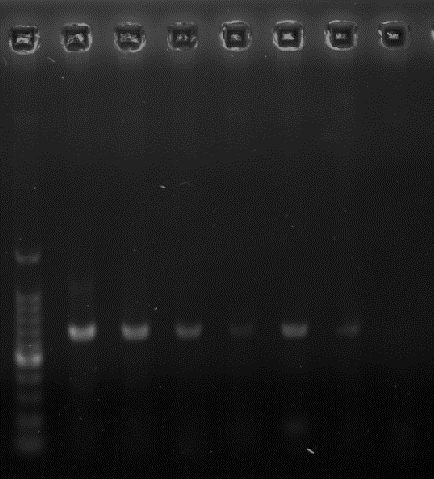  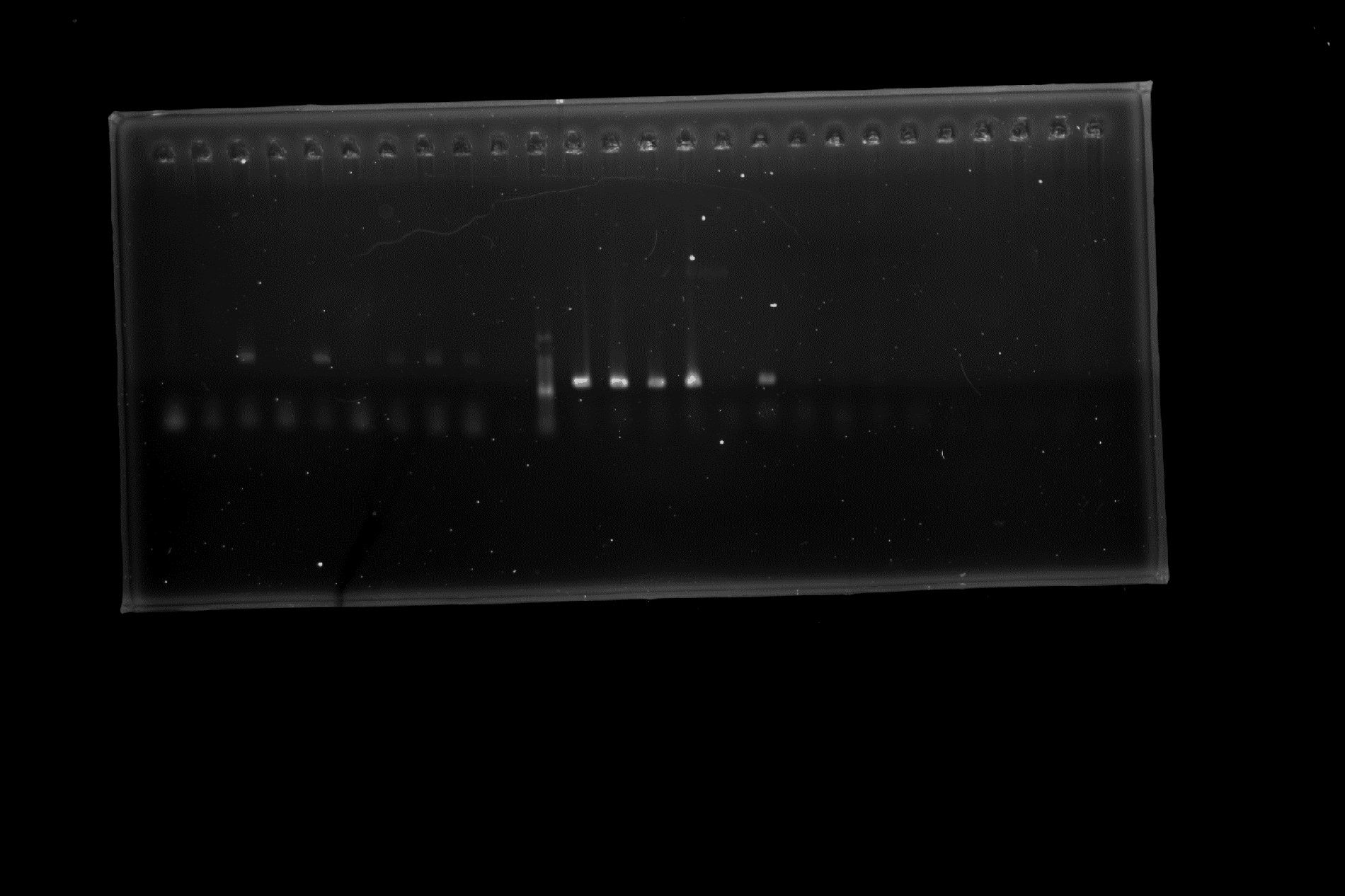  **L 1 2 3 4 N PC**  **L 1 2 3 4 5 PC N**    793 bp  713 bp  **I:** Molecular detection of *bla_TEM_* gene that amplified by 793 bp. Here L represents 100 bp DNA ladder, N negative control, PC- positive control, and 1, 2, 3, 4, represents PCR positive for *bla_TEM_* gene  **H:** Molecular detection of *vanA* gene that amplified by 713 bp. Here L represents 100 bp DNA ladder, N negative control, PC- positive control, and 1, 2, 3, 4, 5, represented PCR positive for *vanA* gene |
| --- |
