# Supplementary figures and images for: Biofilms and antibiotic resistance profile of Enterococcus faecalis in selected dairy cattle farm environments in Bangladesh
Source: PLoS One. 2025 May 19;20(5):e0323667. doi: 10.1371/journal.pone.0323667 (PMC12087997; doi:10.1371/journal.pone.0323667)

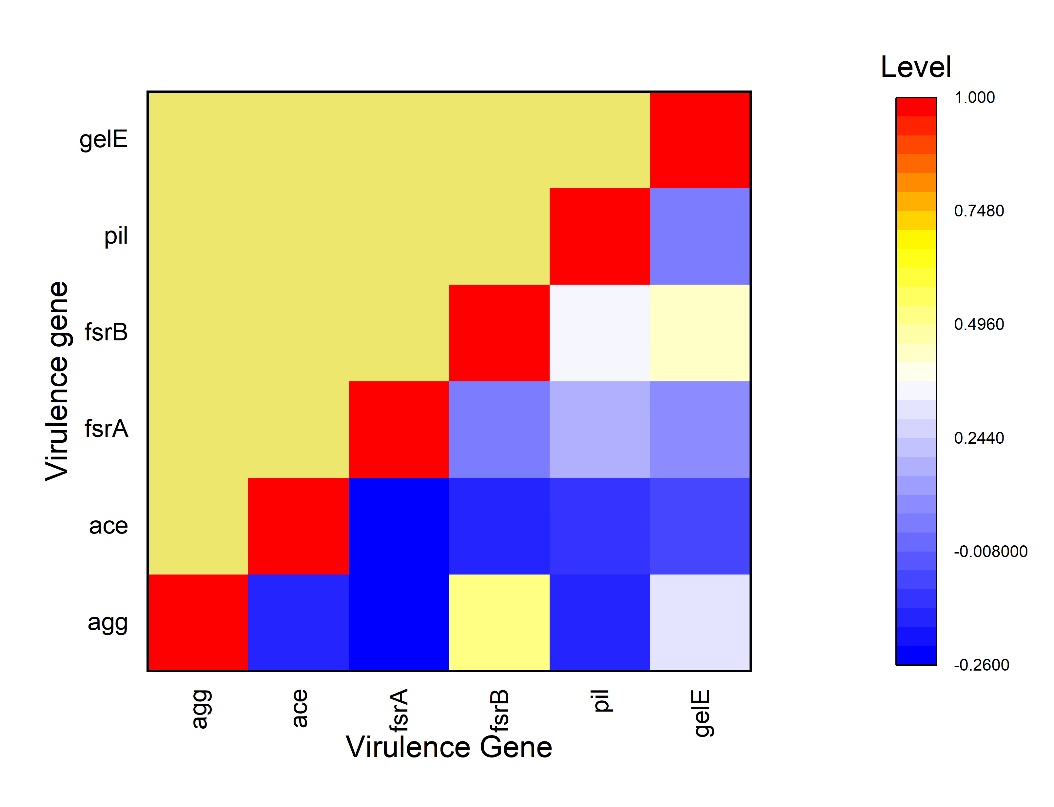


**S2 Fig:** **Heatmap represents the correlation between two virulence genes of E. faecalis**

Supplement: S2 Fig — (DOCX) [file pone.0323667.s002.docx]

| 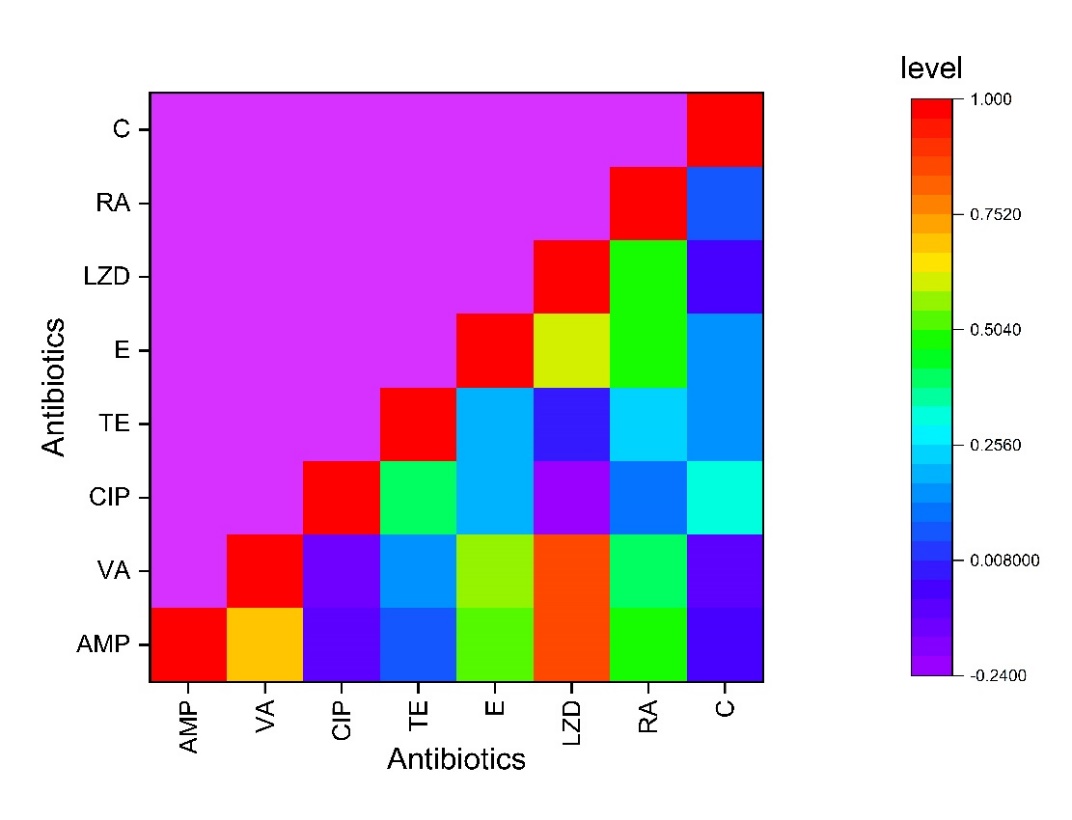  **S3 Fig:** **Correlations between the antibiotic-resistant isolates of E. faecalis** |
| --- |

Supplement: S3 Fig — (DOCX) [file pone.0323667.s003.docx]
