# Supplementary material for: Biofilms and antibiotic resistance profile of Enterococcus faecalis in selected dairy cattle farm environments in Bangladesh
Source: PLoS One. 2025 May 19;20(5):e0323667. doi: 10.1371/journal.pone.0323667 (PMC12087997; doi:10.1371/journal.pone.0323667)
Supplement: S1 Table — (DOCX) [file pone.0323667.s004.docx]

| **Targeted factors** | **Targeted genes** | **Primer Sequences (5′-3′)** | **Annealing Tm (° C)** | **Size (bp)** | **References** |
| --- | --- | --- | --- | --- | --- |
| *E. faecalis* | *ddl_E. faecalis_* | F: ATCAAGTACAGTTAGTCTT | 50 | 941 | (1) |
|  |  | R: ACGATTCAAAGCTAACTG |  |  |  |
| Virulence gene | *agg* | F: TCTTGGACACGACCCATGAT | 58 | 413 | (2) |
|  |  | R: AGAAAGAACATCACCACGAC |  |  |  |
|  | *fsrA* | F: CGTTCCGTCTCTCATAGTTA | 53 | 474 |  |
|  |  | R: GC*AGG*ATTTG*AGG*TTGCTAA |  |  |  |
|  | *fsrB* | F: TAATCT*AGG*CTTAGTTCCCAC | 55 | 428 |  |
|  |  | R: CTAAATGGCTCTGTCGTCTAG |  |  |  |
|  | *gelE* | F: GGTGAAGAAGTTACTCTGAC | 52 | 704 |  |
|  |  | R: GGTATTGAGTTATG*AGG*GGC |  |  |  |
|  | *ace* | F: GAATGACCGAGAACGATGGC | 58 | 615 |  |
|  |  | R: CTTGATGTTGGCCTGCTTCC |  |  |  |
|  | *pil* | F: GAAGAAACCAAAGCACCTAC | 53 | 620 |  |
|  |  | R: CTACCTAAGAAAAGAAACGG |  |  |  |
| Antibiotic resistance gene | *bla_TEM_* | F: CATTTCCGTGTCGCCCTTAT | 56 | 793 | (3) |
|  |  | R: TCCATAGTTGCCTGACTCCC |  |  |  |
|  | *vanA* | F: GGCAAGTCAGGTGAAGATG | 55 | 713 | (4) |
|  |  | R: ATCAAGCGGTCAATCAGTTC |  |  |  |

**S1 Table: List of primers used to find target genes in this investigation**

**References:**

1. Dutka-malen S, Evers S, Courvalin P, et al. Detection of Glycopeptide Resistance Genotypes and Identification to the Species Level of Clinically Relevant Enterococci by PCR. J Clin Microbiol. 1995; 33(1):24-7. doi: 10.1128/jcm.33.1.24-27.1995.
2. Hashem YA, Amin HM, Tamer ME, Yassin AS, Aziz RK, et al. Biofilm formation in enterococci :genotype-phenotype correlations and inhibition by vancomycin. Sci Rep. 2017; 7(1):5733. doi: 10.1038/s41598-017-05901-0.
3. Randall LP, Cooles SW, Osborn MK, Piddock LJ V, Woodward MJ, et al. Antibiotic resistance genes , integrons and multiple antibiotic resistance in thirty-five serotypes of Salmonella enterica isolated from humans and animals in the UK. J Antimicrob Chemother. 2004; 53(2):208-16. doi: 10.1093/jac/dkh070.
4. Azimian A, Havaei A, Fazeli H, Naderi M, Ghazvini K, Samiee M, et al. Genetic Characterization of a Vancomycin-Resistant Staphylococcus aureus Isolate from the Respiratory Tract of a Patient in a University Hospital in Northeastern Iran. J Clin Microbiol. 2012; 50(11):3581-5. doi: 10.1128/JCM.01727-12
