# Supplementary material for: Biofilms and antibiotic resistance profile of Enterococcus faecalis in selected dairy cattle farm environments in Bangladesh
Source: PLoS One. 2025 May 19;20(5):e0323667. doi: 10.1371/journal.pone.0323667 (PMC12087997; doi:10.1371/journal.pone.0323667)
Supplement: S2 Table — (DOCX) [file pone.0323667.s005.docx]

**S2 Table: Pearson correlation coefficient in virulent genes of the isolated *E. faecalis***

| **Correlations** | | | | | | | |
| --- | --- | --- | --- | --- | --- | --- | --- |
|  | | ***agg*** | ***ace*** | ***fsrA*** | ***fsrB*** | ***pil*** | ***gelE*** |
| *agg* | PC | 1 |  |  |  |  |  |
|  | Sig. (2-tailed) |  |  |  |  |  |  |
| *ace* | PC | -0.169 | 1 |  |  |  |  |
|  | Sig. (2-tailed) | 0.430 |  |  |  |  |  |
| *fsrA* | PC | -0.258 | -0.218 | 1 |  |  |  |
|  | Sig. (2-tailed) | 0.223 | 0.306 |  |  |  |  |
| *fsrB* | PC | .507^*^ | -0.143 | 0.073 | 1 |  |  |
|  | Sig. (2-tailed) | 0.011 | 0.505 | 0.736 |  |  |  |
| *pil* | PC | -0.135 | -0.114 | 0.174 | 0.342 | 1 |  |
|  | Sig. (2-tailed) | 0.530 | 0.596 | 0.416 | 0.102 |  |  |
| *gelE* | PC | 0.302 | -0.064 | 0.098 | .447^*^ | 0.051 | 1 |
|  | Sig. (2-tailed) | 0.151 | 0.767 | 0.650 | 0.028 | 0.813 |  |
| *. Correlation is significant at the 0.05 level (2-tailed).  PC= Pearson Correlations | | | | | | | |
