# Supplementary material for: Biofilms and antibiotic resistance profile of Enterococcus faecalis in selected dairy cattle farm environments in Bangladesh
Source: PLoS One. 2025 May 19;20(5):e0323667. doi: 10.1371/journal.pone.0323667 (PMC12087997; doi:10.1371/journal.pone.0323667)
Supplement: S3 Table — (DOCX) [file pone.0323667.s006.docx]

**S3 Table: Pearson correlation coefficient among the *Enterococcus faecalis* resistance isolates**

| Correlations | | | | | | | | | | |
| --- | --- | --- | --- | --- | --- | --- | --- | --- | --- | --- |
|  |  | AMP | VA | CIP | LEV | TE | E | LEN | RA | C |
| AMP | PC | 1 |  |  |  |  |  |  |  |  |
|  | Sig. (2-tailed) |  |  |  |  |  |  |  |  |  |
| VA | PC | .675^**^ | 1 |  |  |  |  |  |  |  |
|  | Sig. (2-tailed) | 0.000 |  |  |  |  |  |  |  |  |
| CIP | PC | -0.086 | -0.125 | 1 |  |  |  |  |  |  |
|  | Sig. (2-tailed) | 0.597 | 0.444 |  |  |  |  |  |  |  |
| LEV | PC | .^b^ | .^b^ | .^b^ | .^b^ |  |  |  |  |  |
|  | Sig. (2-tailed) |  |  |  |  |  |  |  |  |  |
| TE | PC | 0.089 | 0.154 | .385^*^ | .^b^ | 1 |  |  |  |  |
|  | Sig. (2-tailed) | 0.583 | 0.342 | 0.014 |  |  |  |  |  |  |
| E | PC | .507^**^ | .586^**^ | 0.192 | .^b^ | 0.200 | 1 |  |  |  |
|  | Sig. (2-tailed) | 0.001 | 0.000 | 0.234 |  | 0.216 |  |  |  |  |
| LEN | PC | .843^**^ | .850^**^ | -0.238 | .^b^ | 0.000 | .589^**^ | 1 |  |  |
|  | Sig. (2-tailed) | 0.000 | 0.000 | 0.139 |  | 1.000 | 0.000 |  |  |  |
| RA | PC | .488^**^ | .383^*^ | 0.126 | .^b^ | 0.218 | .480^**^ | .463^**^ | 1 |  |
|  | Sig. (2-tailed) | 0.001 | 0.015 | 0.439 |  | 0.176 | 0.002 | 0.003 |  |  |
| C | PC | -0.059 | -0.086 | 0.306 | .^b^ | 0.132 | 0.132 | -0.047 | 0.087 | 1 |
|  | Sig. (2-tailed) | 0.717 | 0.599 | 0.055 |  | 0.415 | 0.415 | 0.774 | 0.595 |  |

PC= Pearson Correlations

**. Correlation is significant at the 0.01 level (2-tailed).

*. Correlation is significant at the 0.05 level (2-tailed).

b. Cannot be computed because at least one of the variables is constant.

**Legends**, AMP= Ampicillin; C= Chloramphenicol; LZD= Linezolid; CIP= Ciprofloxacin; E= Erythromycin; RA= Rifampicin; TE= Tetracycline; LEV= levofloxacin and VA = Vancomycin
