# Supplementary material for: Biofilms and antibiotic resistance profile of Enterococcus faecalis in selected dairy cattle farm environments in Bangladesh
Source: PLoS One. 2025 May 19;20(5):e0323667. doi: 10.1371/journal.pone.0323667 (PMC12087997; doi:10.1371/journal.pone.0323667)
Supplement: S4 Table — (DOCX) [file pone.0323667.s007.docx]

**S4 Table: Mapping of multi-drug-resistant *E. faecalis* isolates**

| **Types** | **Categories (N)** | **Occurrence of multidrug-resistant isolates n (%) ^S^** | **95% CI (%)** | *p*-value |
| --- | --- | --- | --- | --- |
| Samples type | Floor Surface (9) | 6 (66.67 ^a^) | (35.42-87.94) | 0.810 |
|  | Feces (5) | 4 (80 ^a^) | (37.55-96.38) |  |
|  | Feed (7) | 4 (57.14 ^a^) | (25.05-84.18) |  |
|  | Manure (8) | 7 (87.5 ^a^) | (52.91-97.76) |  |
|  | Drainage Water (5) | 3 (60 ^a^) | (23.07-88.24) |  |
|  | Drinking Water (6) | 4 (66.67 ^a^) | (30.00-90.32) |  |
|  | Total (40) | 28 (70) | (54.57 – 81.93) |  |
| Locations | Ullapara, Sirajganj (13) | 13 (100 ^a^) | (77.19 – 1.00) | 0.040 |
|  | Digarkanda, Mymensingh (11) | 7 (63.64 ^b^) | (35.38 – 84.83) |  |
|  | Boyra, Mymensingh (13) | 8 (61.54 ^b^) | (35.52 – 82.29) |  |

Here, Within the variable being evaluated, S=values with different superscripts differ significantly (p < 0.05), N = number of isolates sampled by category, n = No of isolates that showed MDR, CI = confidence interval
